# Protective effect of ethyl pyruvate on mice sperm parameters in phenylhydrazine induced hemolytic anemia

**Published:** 2016-03-15

**Authors:** Ali Akbar Mozafari, Rasoul Shahrooz, Abbas Ahmadi, Hassan Malekinjad, Karim Mardani

**Affiliations:** 1 *Department of Basic Sciences, Faculty of Veterinary Medicine, Urmia University, Urmia, Iran;*; 2 *Department of Food Hygiene and Quality control, Faculty of Veterinary Medicine, Urmia University, Urmia, Iran.*

**Keywords:** Ethyl pyruvate, Mouse, Phenylhydrazine, Sperm parameters, Stress oxidative

## Abstract

The aim of the present study was to assess the protective effect of ethyl pyruvate (EP) on sperm quality parameters, testosterone level and malondialdehyde (MDA) in phenylhydrazine (PHZ) treated mice. For this purpose, 32 NMRI mice with the age range of 8 to 10 weeks, weight average 26.0 ± 2.0 g, were randomly divided into four equal groups. The control group (1) received normal saline (0. 1 mL per day) by intraperitoneal injection (IP). Group 2 (PHZ group) was treated with initial dose of PHZ (8 mg 100 g^-1^, IP) followed by 6 mg 100 g^-1^ , IP every 48 hr. Group 3, (Group PHZ+EP) received PHZ (according to the previous prescription) with EP (40 mg kg^-1^, daily, IP). Ethyl pyruvate group (4) received only EP (40 mg kg^-1^, daily, IP). Treatment period was 35 days. After euthanasia, sperms from caudal region of epididymis were collected and the total mean sperm count, sperm viability, motility and morphology were determined. Testis tissue MDA and serum testosterone levels of all experimental groups were also evaluated. A considerable reduction in mean percentage of number, natural morphology of sperm, sperm motility and viability and serum testosterone concentration besides DNA injury increment among mice treating with PHZ in comparison with control group were observed. However, in PHZ+EP group the above mentioned parameters were improved. This study showed that PHZ caused induction of toxicity on sperm parameters and reduction of testosterone as well as the increment of MDA level and EP as an antioxidant could reduce destructive effects of PHZ on sperm parameters, testosterone level and lipid peroxidation.

## Introduction

In recent years antioxidant products have gained attention based on their protective effects against drugs–induced toxicities, especially whenever free radical generation is involved.^1^ The antioxidant compound(s) intake in human diet that enhance the biological anti-oxidant mechanism can prevent and in some cases helps in the treatment of some oxidative–related disorders and organ toxicity events.^2^

The hemolytic activity of phenylhydrazine (PHZ)**, **may lead to acute hemolytic anemia in vertebrates.^3^ The main action PHZ has long been associated with drug-induced stress oxidative occurring within erythrocytes.^4^ The PHZ is an antipyretic drug that was first characterized by Emil Fisher in 1875. This drug is well known for its ability to produce hemolysis in rats and humans. 

Phenylhydrazine has long been known as a hemolytic agent^5^ and historically it was used as a therapeutic agent in the treatment of polycythemia vera. The mechanism of action of this hydrazine has been extensively studied.^6^ The interaction of PHZ and hemoglobin generates hydrogen peroxide and destroys the pigment through the formation of oxidative derivatives and free radicals of the hydrazine.^7^ Phenylhydrazine is capable of inhibiting the function of liver microsomal cytochrome P-450 through free radicals formation.^8^

 Pyruvate is a key intermediate metabolite of glucose, a potent antioxidant and free radical scavenger.^9^ Pyruvate has been shown to afford protection in numerous *in vitro* and *in vivo* models including oxidant mediate cellular or organ system injury.^10^ Pyruvate plays a crucial part in intermediary metabolism being the final product of glycolysis and the prominent substrate for tricarboxylic acid (TCA) cycle, and it probably acts as an endogenous antioxidant in cells.^11^ Despite these promising findings**, **the usefulness of pyruvate as a therapeutic agent is limited by its poor stability insulation. Aqueous solutions of pyruvate rapidly undergo a spontaneous aldol-like condensation reaction to form 2-hydroxy-2-methyl-4-ketoglutarate**, **a compound that also is known as parapyruvate.^12^

Pyruvate has been reported to be an effective scavenger of reactive oxygen species (ROS) in cell as well as an anti-inflammatory agent.^13^ In spite of the antioxidant activity of seminal plasma, epididymis and spermatozoa damage sperm functions and DNA integrity.^14^ The main anti-oxidative defense in seminal plasma includes SOD, GPx, vitamin E , vitamin C.^15^ Koksal *et al*. demonstrated that severe pathologic changes in testicular tissue are associated with high level of lipid peroxidation and suggested that overproduction of ROS may play a role in the mechanism of testicular degeneration associated with infertility.^16^

The present study was conducted to evaluate the protection role of ethyl pyruvate (EP) on stress oxidative induced by PHZ in sperm quality.

## Materials and Methods

**Chemicals. **The EP (E47808) and PHZ (P6926) were purchased from Sigma-Aldrich (St. Louis, USA), which was used as the test substance. 

**Animals and treatment groups. **Adult male NMRI mice with the age range 8 to 10 weeks old and average weight 26.0 ± 2.0 g were purchased from animal house of Faculty of Veterinary Medicine, Urmia University, Urmia, Iran. Animals were kept under a controlled environmental condition at room temperature (22.0 ± 2.0 ˚C) with humidity of 5 ± 10% and a 12/12 hr photoperiod. The animals were nourished with standard pellets and water ad libitum. Thirty two animals were randomly divided into four equal groups. In group 1, control group received intraperitoneal (IP) normal saline (0.1 mL per day). Mice in Group 2, PHZ group, received PHZ with initial dose of (8 mg 100 g^-1^, IP) followed by sequential dose 6 mg per 100 g, every 48 hr, IP.^17^ Group 3, PHZ+EP group, received PHZ the same as second group along with EP at dose of 40 mg kg^-1^,every day, IP. Group 4, EP group, received only EP at the same dose like the third group. The animals were treated for 35 days. After 35 days, the mice were anesthetized by 25 mg kg^-1^ ketamine (Alfasan, Utrecht, The Netherlands) for blood sampling and they were euthanized with ketamine over dose (100 mg kg^-1^). Then both epididymis (caudal region) of each mouse were transferred to a 60 mm petri dish containing 1 mL human tubal fluid (Sigma-Aldrich) cultured and 4 mg mL^-1^ bovine serum albumin (Sigma-Aldrich) medium pre-heated at 37 ˚C. The caudate of epididymis was minced making five to seven slashes with a 30-gauge needle. After 30 min incubation at 37 ˚C in 5% CO_2_, spermatozoa released from epididymis.

**Assessment Sperm count. **In order to count sperms a 1:20 dilution prepared in a 1 mL micro tube 190 µL of distilled water was poured and 10 µL of sperm mixture was added to it. Then, 1 µL of the mixture was dropped on a Neobar slide and the sperm were counted.^18^

**Sperm viability.** Sperm viability was evaluated as follows. Volume of 20 µL of 0.05% Eosin Y-nigrosin was added into an equal volume of sperm suspension. After2 min of incubation at room temperature, slides were observed by a light microscope with magnification of 400×. Dead sperms were stained pink but the live ones took no color. Sperms (n = 400) were counted in each sample and the viability percentage was computed.^19^

**Evaluating sperm motility.** In order to evaluate sperm motility, 10 µL sperm suspension was placed on a pre-heated slide covered with a slip and then the motility was observed by under a light microscope (Nikon, Tokyo, Japan) with 400× magnification.^20^

**Sperm morphology.** To evaluate sperm morphology in the present study, aniline blue (AB) staining method was implemented and abnormal morphologies percentage was determined. Especially, the cytoplasmic residual of sperms was considered as abnormal morphology.^21^

**Determining damage to DNA.** Fragmentation of sperm DNA was applied as a biomarker for male infertility. Acridine orange (AO) staining was used, after challenging at low pH, to distinguish between denatured, native, and double-stranded, DNA regions in sperm chromatin.^22^ Results indicated high level of fluorescent in denatured DNA. Thick smears were placed in carnoy’s fixative (methanol/acetic acid 1:3) for 2 hr for fixation.^23^ The slides were removed from the fixative and they were left on the outside to be dried for 5 min at laboratory temperature. Then, slides were placed in a stock solution of 1 mg of AO in 1000 mL distilled water and stored in a dark place at 4 ˚C. The stained solution was produced then, contained 10 mL of the stock which was added to 40 mL 0.3 M NaHPO_4_.7H_2_O solution.^24^ After 5 min staining, sperms were examined using fluorescent microscope. Green colored sperms were observable among normal sperms and yellow-red sperms were categorized in abnormal or damaged DNA.^25^

**Evaluating sperm nucleus maturity.** During the replacement of histone with protamine, and condensation of chromatin in spermiogenesis, normal sperms were not stained with AB while abnormal sperms, after condensation, or immature sperm got stained easily.^27^ Sperm samples were fixed for 30 min in glutaraldehyde 3% in saline phosphate buffer (pH = 7.2). Each smear was stained with AB. At least 200 spermatozoa using light microscopy (Olympus, Tokyo, Japan) were counted in each slide.^26^

**Measurement of malondialdehyde (MDA). **A volume of 300 µL of 10% trichloro-acetic acid (Sigma-Aldrich) was added to 150 µL of the sample and centrifuged at 1000 rpm for 10 min at 4 ˚C, then incubated in 300 µL of 67.0% thio-barbituric acid (TBA) at 100 ˚C for 25 min. Five min after cooling the solution, pink color appeared because of MDA-TBA reaction and was evaluated using a spectrometer (Novaspec II; Biochrom Ltd., Cambridge, UK) at a wave length of 535nm.^27^

**Testosterone assay. **The mice were anesthetized for blood sampling. After collecting blood samples they were centrifuged for isolation of serum and kept at – 80 ˚C until biochemical analysis with immune-radiometric technique (WHO/Sigma Asso-RTGC-768/98) for testosterone measurement (Abbott Laboratories, Abbott Park, USA).

**Statistical analysis. **Results were shown as mean ± standard error mean (SEM) for each group, to compare the groups. Results were analyzed by SPSS (Version 16; SPSS Inc., Chicago, USA), using one-way ANOVA followed by a Bonferroni post hoc test. All data were evaluated at *p* < 0.05 levels and were considered significant.

## Results

**Average sperm count. **The results revealed that the number of sperms was decreased significantly (*p *< 0.05) in PHZ group in comparison with the control group. Group 3, revealed significant enhancement compared to PHZ group (*p* < 0.05). There was no significant difference in sperm count in EP group comparing the control groups (Table 1). 

**Fig. 1:**
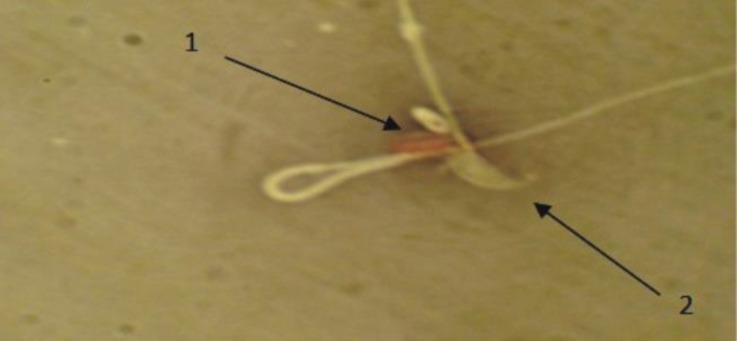
Evaluation of spermatozoa viability. **1)** Dead sperms with red heads (dark); **2)** Alive sperms with heads (bright), (Eosin Y-nigrosin, 1000×).

**Viability power of sperm.** The comparison of sperm viability average in PHZ group to control group showed that it was significantly reduced (*p *< 0.05), (Table 2). However, with administration of antioxidant in PHZ+EP group, this reduction of sperm viability was improved, but there was significant difference (*p* < 0.05) in control and PHZ groups. The EP group had only a significant difference with PHZ group (*p *< 0.05), (Fig. 1 and Table 1).

**Fig. 2:**
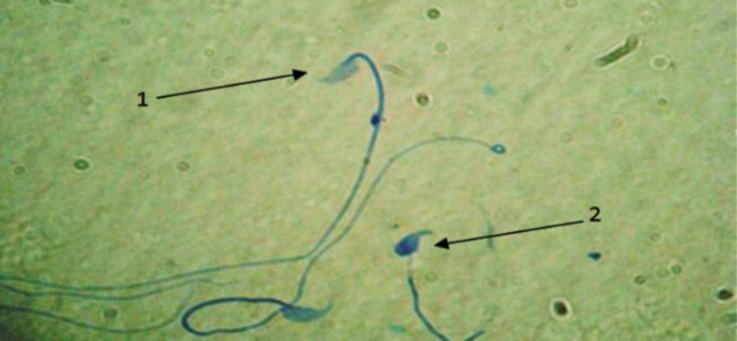
Maturity evaluation of spermatozoa. **1)** Sperms with mature nucleus show heads in pale blue; **2)** Immature nucleus sperms represents dark blue head, (Aniline blue, 1000×).

**Sperm nucleus immaturity. **Monitoring the immaturity of sperm nucleus in PHZ group compared to the control group indicated that it was increased significantly (*p *< 0.05). Administration of antioxidant in EP+PHZ group and EP group the amount of the sperm with immature nucleus was lowered, however, there was a significant difference between control and PHZ groups (*p* < 0.05). However, EP showed significant difference in comparison with PHZ group (*p *< 0.05), (Fig. 2 and Table 1). 

**Sperm mobility status. **Comparison of sperm mobility average in PHZ group to control group showed significant decrease (*p* < 0.05), (Table 2). However, with antioxidant administration in PHZ ± EP this reduction of mobility was improved, but in control and PHZ groups there was a significant difference (*p* < 0.05). The EP group showed significant difference with PHZ group only (*p* < 0.05), (Table 1). 

**Fig. 3:**
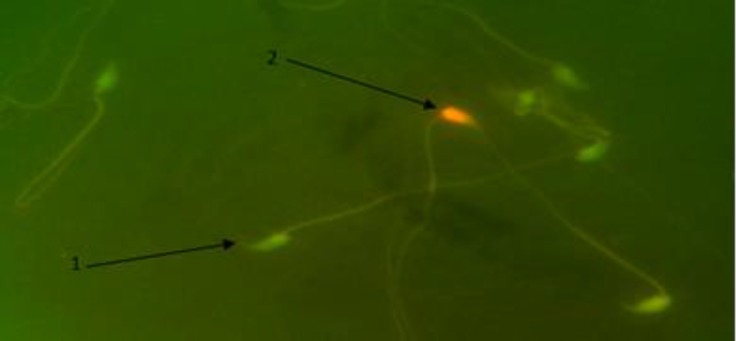
Detection of DNA damage in spermatozoa. **1)** DNA of healthy sperms have green heads; **2)** DNA of damaged sperms heads are in red color (Acridine orange, 1000×).

**Sperm DNA damage. **To study sperm DNA damage, the AO staining method was used. The percentage of sperm with damaged DNA in PHZ group was increased compared to the control group, (*p* < 0.05). However, in PHZ+EP group it was reduced in comparison with PHZ group, yet it was significantly higher than the control group (*p* < 0.05). The percentage of sperm with damaged DNA in EP group was almost the same as the control group, (Fig. 3 and Table 1). 

**Sperm morphology. **The mean percent of sperm with normal morphology in PHZ group was significantly lower than the control group, but with administration of EP in EP+PHZ group this reduction was improved a little, but that was significantly low compared to PHZ group (*p* < 0.05). Also, in EP group, in spite of high percentage of normal sperm, it was significantly different from the control and PHZ groups (*p* < 0.05), (Table 1). 

**Table 1:** The protective effect of ethyl pyruvate (EP) and phenylhydrazine (PHZ) on sperm parameters

**Groups**	**Sperm count (×10** ^6^ **)**	**Sperm motility (%)**	**Sperm morphology (%** **)**	**Sperm viability (** **%)**	**Immature sperm (%** **)**	**DNA damage (%)**
**Control**	26.25	58.25 ± 3.03	75.50 ± 1.04	71.60 ± 2.85	2.75 ± 0.62	1.25 ± 0.47
**PHZ**	16.25^a^	33.50 ± 3.27^a^	56.00 ± 1.08^a^	31.40 ± 3.00^a^	30.50 ± 1.32^a^	43.00 ± 2.19^a^
**PHZ+EP**	22.75^b^	50.75 ± 2.86^ab^	61.25 ± 1.65^b^	61.60 ± 3.02^b^	8.50 ± 1.04^ab^	27.75 ± 2.13^ab^
**EP**	25.06^b^	61.50 ± 4.62^b^	69.75 ± 0. 85^ab^	68.80 ± 2.51^b^	3.25 ± 0.75^bc^	3.00 ± 1.08^bc^

abc Different superscripts indicate significant difference among the groups in each column (*p* < 0.05).

**Results of MDA and testosterone evaluating.** The level of MDA, as a major product of lipid peroxidation was significantly increased in PHZ group compared to the control group (*p *< 0.05). There was a significant restoration of MDA level in PHZ+EP and EP groups. The level of testosterone was found to be significantly lower in PHZ group in comparison with other groups (*p *< 0.05). 

In PHZ+EP group there was a significant increase in testosterone concentration compared to PHZ and control groups (*p* < 0.05). Testosterone level in EP group was higher than that in the others (*p *< 0.05), (Table 2). 

**Table 2:** Comparison of the effect of ethyl pyruvate (EP) on malondialdehyde and testosterone levels affected by phenyl-hydrazine (PHZ

**Groups**	**Malondialdehyde (µmol g** ^-1^ ** of tissue)**	**Testosterone (ng mL** ^-1^ **)**
**Control**	3.74 ± 0.58	7.16 ± 0.48
**PHZ**	9.41 ± 0.45^a^	4.52 ± 0.58^a^
**PHZ+EP**	4.75 ± 0.56^ab^	5.17 ± 0.47^ab^
**EP**	4.25 ± 0.44^ab^	7.83 ± 0.39^ab^

abc Different superscripts indicate significant difference among the groups in each column (*p* < 0.05).

## Discussion

The oxidative status of cells is determined by the balance between pro-oxidants and antioxidants. Pro-oxidants, referred to as ROS, are classified into radicals and non-radicals.^28^ Free radicals are molecules with one or more unpaired electrons.^29^ In experiments with PHZ, it was observed that hydrazine has the ability to generate superoxide anion radical and hydrogen peroxide to cause the formation of lipid peroxidation and Heinz body formation.^30^ Also, the present study showed that there was a clear relationship between ROS production and apoptosis, causing DNA damage in sperms. These subjects were accommodated with the observations of this study related to effects of PHZ that caused a significant increase DNA fragmentation (*p* < 0.05). However, the present study revealed that the sperm count was diminished significantly in PHZ group in comparison with control group, whereas in PHZ+EP group it was elevated compared to PHZ group (*p *< 0.05). The results of the present study also revealed that PHZ induced stress oxidative on cells of spermato-genesis and it was prevented with EP. Sperm count was crude measure of fertility.^31^

The present study revealed that the sperm count was diminished significantly in PHZ group compared to the control group whereas in PHZ+EP group it was elevated compared to PHZ group. EP group only showed a significant difference with PHZ group (*p *< 0.05). 

Decrease in sperm numbers often results from the interference in spermatogenesis process and elimination of sperm cells at different stages of development.^32^ This reduction maybe related to increased production of free radicals due to PHZ administration that in turn causes disorders in functionality of Leydig and Sertoli cells.^33^ In Leydig cells, increase in oxidative stress causes a decline in synthesis and secretion of testosterone. Reduction of this hormone is an effective factor to cause disorder in spermio-genesis and decrease in epididymal sperm count.^34^ Thus, increase in stress oxidative elevate the free radicals concentration and on the other hand causes decrease in the antioxidant level in the Leydig cells. Our results in sperm counts were in agreement with previous studies.^35^ Sperm motility is also as important as the counts in male fertility issue. The comparison of the sperm mobility average in PHZ group with control group showed significant reduction that improved the mobility reduction with administration of EP+PHZ, however, between control and PHZ groups there was a significant difference (*p *< 0.05). The EP group showed significant difference compared to PHZ group. Therefore, antioxidant not only prevents decrease of the sperm motility, however, causes increase of its capability.^36^^,^^37^ The ROS overproduction may cause peroxidation of sperm cell membrane lipids affecting structure of enzymes, receptors and transport of proteins.^38^ Lipid peroxidation triggers the loss of membrane integrity and results in the increased cell electrolyte permeability. This may affect cellular energy metabolism and cause the depletion of ATP and result in loss motility and viability of sperms.^39^

Therefore, increase in free radical causes peroxidation of sperm cell membrane lipid and loss of sperm membrane integrity. Results showed that the number of sperms with abnormal shape and morphology in PHZ group had significant reduction compared to the control group and with administration of antioxidant in EP+PHZ group the count of sperms were decreased, however, yet it had a significant difference with control group. Therefore, it can be said that sperm disorder induced by PHZ due to histologic, cytotoxic and genetic changes in DNA causes an increase in the number of abnormal sperms.^40^

Our results in sperm morphology were in agreement with aforementioned studies. Lipid peroxidation products in testis were determined by measuring MDA. Assay of MDA is a good indicator of the degree of lipid peroxidation and in the present study the level of MDA in PHZ group was significantly higher than the control group. Administration of EP along with PHZ were decreased in level of MDA, hence, it was significantly higher than the control group. Malondialdehyde molecule caused asymmetry in distribution of lipid components of membranes via interfering with the membrane and ends up injury and breakage of chromosomes through creating strong link with DNA of the cell.^41^ Our results in MDA measurement were in agreement with aforementioned studies.

The amount of testosterone level in PHZ group compared to control group showed a significant difference reducing with administration of EP, however, it was still significant compared to control and PHZ group. Testis tissue damages are created by oxidative stress from PHZ that causes the creation of free radicals which in turn cause the creation of sperm wall, as well as interstitial cells, unsaturated lipid peroxidation and destruction. Because of the reduction in interstitial cells number, the amount of testosterone was decreased and improved with administration of anti-oxidant and resulted in reduction in free radicals numbers. Testosterone is essential to maintain the structure and function of the male accessory sex gland. Moreover, lack of testosterone disrupts spermatogenesis.^42^ EP is unlikely to be harmful to humans, given its close similarity to an endogenous metabolite, its safety profile in animals and its common use as a food supplement. The EP can react with ROS both via oxidative carboxylation and formation of hydroxylated adducts at the 3-carbon.^43^

It could be concluded that EP as a non-enzymatic antioxidant could protect the reproductive organs against adverse effects of hemolytic anemia induced by PHZ. The present study showed that EP through scavenging of free radicals could reduce the adverse effects of hemolytic anemia in sperm, such as DNA damage, low sperm counts and decrease in viability of sperm and low level of testosterone as well as high level of MDA in mice. 
